# Enhanced Range Resolution Beamforming for Subarray-Based FDA

**DOI:** 10.3390/s26072104

**Published:** 2026-03-28

**Authors:** Anyi Wang, Yumeng Lu, Yanhong Xu

**Affiliations:** Xi’an Key Laboratory of Network Convergence Communication, College of Communication and Information Engineering, Xi’an University of Science and Technology, Xi’an 710054, China; wanganyi@xust.edu.cn (A.W.); 23207040040@stu.xust.edu.cn (Y.L.)

**Keywords:** transmit-receive beamforming, subarray-based frequency diverse array, range resolution, BA, K-means++ clustering algorithm

## Abstract

To address the range-angle coupling issue of frequency diverse array (FDA), a beamforming method based on subarray partitioning is proposed, with a focus on analyzing uniform continuous and nonuniform discontinuous subarray structures. Based on the transmit–receive signal model established to solve the time-varying issue of FDA, two subarray partitioning methods under the same array aperture are investigated. In the case of uniform continuous subarray structure, when different linear frequency offsets (FOs) are applied to each subarray, the mainlobe width in range dimension is 4.35 km, and the peak sidelobe level (PSLL) is −7.25 dB. When nonlinear FOs are applied, the mainlobe width is reduced to 2.76 km, and the PSLL is decreased to −9.64 dB. Furthermore, by adopting a nonuniform discontinuous subarray structure combined with nonlinear FOs, the mainlobe width is further narrowed to 1.29 km, and the PSLL is reduced to −11.75 dB. The simulation results demonstrate that under the same conditions, the nonuniform discontinuous subarray structure significantly improves range resolution and effectively suppresses sidelobe. Based on above results, a joint optimization combining the bat algorithm (BA) and K-means++ clustering algorithm is proposed to optimize the subarray structure and element amplitudes simultaneously. Finally, the mainlobe width of the optimized FDA is 1.18 km and the PSLL is −12.32 dB. Simulation results confirm the effectiveness and potential of the proposed method in enhancing range resolution and achieving a focused beampattern.

## 1. Introduction

Phased array (PA) is widely used in radar, navigation, and wireless communication systems due to the flexible electronic beam-scanning capabilities. However, the beampattern is solely dependent on angle, meaning the beam points in the same direction across the entire range dimension, making it difficult to directly suppress range-related interference through beamforming [1]. To address this issue, Antonik et al. first introduced the concept of frequency diverse array (FDA) [2]. By introducing small frequency offset (FO) across the array aperture, FDA can generate beampatterns that are simultaneously dependent on both angle and range [3], enabling more flexible beam scanning and control [4]. This characteristic allows FDA to demonstrate significant potential in various range-dependent applications, such as target detection [5,6], range–angle imaging [7,8], wireless power transmission [9,10], synthetic aperture radar (SAR) imaging [11,12], and secure communication [13,14,15,16,17]. As a result, FDA is considered an effective extension of traditional PA and has become one of the key research topics in the field of array signal processing.

However, in FDA, the phase of each array element accumulates with time over the signal duration [18], thereby endowing the FDA with a time-varying characteristic. On the one hand, this characteristic enables the FDA to realize continuous electromagnetic beam scanning during the signal duration. On the other hand, it increases the difficulty of mainlobe control. To alleviate this problem, Khan et al. [19] proposed a time-varying FO which is designed for the array elements to produce a beampattern peak that persists over the entire pulse at a specific range–angle pair, thereby effectively suppressing range-dependent interference and enhancing target detection capability. Yao et al. [20] combined time-domain modulation with nonlinear FOs to realize a time-invariant transmit range-angle beampattern that has a single maximum at the target location. The above works only considered the signal propagation process, and the inherent time-varying problem of the FDA still exists [21]. In this context, researchers began to consider receive-side equivalent transmit beamforming. In [22], Xu et al. proposed a new joint transmit–receive method that processes the time-varying terms through a series of mixers and filters to produce a quasi-static beampattern, whereas [23] further simplified the architecture by adopting only one mixer and one analog-to-digital converter (ADC), significantly reducing the system cost.

Additionally, due to the linear dependence between the linear FO and the element spacing, the conventional linearly spaced FDA, termed as linear-FDA, suffers from range–angle coupling, which causes its beampattern to exhibit mainlobes at multiple ranges and angles simultaneously [24], thereby introducing range ambiguity of the beampattern and interfering with target detection and localization. Consequently, extensive research has been conducted on FDAs employing different types of nonlinear FOs and array configurations. Khan et al. [25] are the first to propose a nonlinear FO based on a natural logarithmic distribution, which can form a single-mainlobe beampattern in the range–angle dimension. However, this method exhibits low range resolution and high peak sidelobe level (PSLL), limiting its practical focusing performance. Since then, various nonlinear FOs have been proposed, including tangent hyperbolic function FO [26], segmented triangular FO [27], symmetric logarithmic FO [28], random logarithmic FO [29], sinusoidal FOs [30,31], polynomial-fitted FO [32], window-function-based FOs [33,34,35,36], as well as other nonlinear FOs obtained by optimization algorithms [37,38,39]. Meanwhile, to investigate the impact of array configuration on FDA beampattern performance, semicircular array configuration [40], circular array configuration [41], arc array configuration [42] and concentric circular array configuration [43] have also been widely studied.

Subarray partitioning method divides the whole array into multiple subarrays and preserves the original array geometry, effectively breaking the linear dependence between the linear FO and the element spacing, thereby enhancing the flexibility of beampattern design and enabling decoupling in the range–angle dimension. In recent years, some studies have focused on the subarray partitioning method of FDA. In [44], an FDA architecture based on two uniform subarrays has been proposed, in which different subarrays adopt different FOs to generate a focused beampattern that permits direct estimation of target range and angle. Xu et al. [45] partitioned the FDA into multiple non-overlapping subarrays and assigned distinct FOs to each subarray so as to obtain degrees of freedom simultaneously in range and angle, thereby achieving effective concentration of the transmit energy within a desired range–angle dimension. Wang et al. [46] further proposed a cross-subarray FDA structure that divides odd- and even-indexed elements into different subarrays and introduces nonlinear FOs, significantly improving transmit-energy focusing capability, sidelobe suppression, and array resolution. Subsequent studies by the same team further explored an FDA employing logarithmic FO with overlapping subarrays, as well as subarray-partitioning designs of planar FDAs [47]. In [48], a configuration with equal frequency within each subarray and linear FOs across subarrays has been adopted, effectively enhancing range resolution. The aforementioned studies did not consider the time-varying issue of the FDA; moreover, in their partitioning schemes, the element spacing within each subarray remains uniform, and thus the linear dependence persists. To address these issues, under a joint transmit–receive signal model, this paper conducts a series of in-depth studies of subarray partitioning methods and proposes a nested optimization algorithm that combines the bat algorithm (BA) with the K-means++ clustering algorithm to simultaneously optimize the subarray structure and the element amplitudes. The main contributions are summarized as follows.

(1) A uniform continuous subarray partitioning method of linear-FDA under a joint transmit–receive signal model is investigated, in which different subarrays employ different FOs. By comparing the beampatterns produced by linear FOs and nonlinear FOs, it can be concluded that FO has significant influence on the performance of FDA.
(2) A nonuniform and discontinuous subarray structure based on the K-means++ clustering algorithm of FDA is proposed. By breaking the traditional continuous subarray structure, the performance limitations have been overcome. Different subarrays adopt different types of nonlinear FO. Simulation results indicate the mainlobe width in range dimension is 1.29 km and the PSLL is -11.75 dB, demonstrating the enhancement in range resolution.
(3) An optimization algorithm that integrates the BA with the K-means++ clustering algorithm is designed to achieve joint optimization of the nonuniform discontinuous subarray structure and the element amplitudes. Simulation results show that the mainlobe width in range dimension is further decreased to 1.18 km and the PSLL is reduced to -12.32 dB. The proposed optimization algorithm can achieve the best performance in terms of range resolution and sidelobe suppression.

The rest of the paper is organized as follows. In Section 2, the transmit–receive beamforming signal models are established for uniform continuous and nonuniform discontinuous subarray structures. In Section 3, a joint optimization algorithm integrating the BA and K-means++ clustering algorithm is proposed to co-optimize the subarray structure and the element amplitudes. Section 4 presents the simulation results and analysis in detail to demonstrate the effectiveness of the proposed method. Finally, Section 5 summarizes the work and presents the conclusions.

## 2. Transmit–Receive Beamforming Signal Models of Subarray-Based FDA

A linear-FDA is considered in this section. The transmit–receive signal models of linear-FDA with uniform continuous subarray structure (Section 2.1) and nonuniform discontinuous subarray structures (Section 2.2) are derived and analyzed.

### 2.1. Transmit–Receive Signal Model of Uniform Continuous Subarray-Based FDA

**Figure 1:**
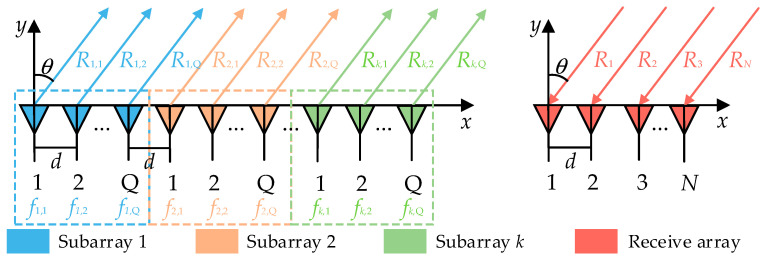
Configuration of the uniform continuous subarray-based FDA.

As depicted in Figure 1, there is a collocated transmit–receive linear-FDA consisting of *M* transmit elements and *N* receive elements with element spacing of d. The transmit array is partitioned into K uniform continuous subarrays $\left(K<M\right)$, each containing Q elements. Within each subarray, the inter-element frequency offset Δf is identical, i.e., the frequencies vary uniformly within the subarray.

The frequency of the *q*th element in the *i*th subarray can be written as

(1) $$f_{i,q}=f_{0}+\left(q-1\right)\Delta f_{i}q=1,2,\ldots ,Qi=1,2,\ldots ,K$$

where $f_{0}$ represents the carrier frequency and $\Delta f_{i}$ is the FO corresponding to the *i*th subarray. Note that $f_{i,1}=f_{0}$. Assume that the distance from the first element (the leftmost element) to the far-field target is R; then the wave path from the *q*th element in the *i*th subarray to the target can be expressed as

(2) $$R_{i,q}=R-\left[\left(i-1\right)Q+q-1\right]d\sin\left(\theta \right)$$

Set $m=\left(i-1\right)Q+q$. Then, Equation (2) can be rewritten as $R_{m}=R-\left(m-1\right)d\sin\left(\theta \right)$. The array geometry remains unchanged before and after subarray partitioning method, and only the FO between adjacent array elements changes. Consequently, the phase difference between the *m*th element signal and the first element signal can be expressed as

(3) $$\Delta \psi_{m}=\psi_{m}-\psi_{1}=2\pi \left[\left(q-1\right)\Delta f_{i}\left(t-\frac{R}{c}\right)-\frac{f_{0}\left(m-1\right)d\sin\left(\theta \right)}{c}\right]$$

where $\psi_{m}=2\pi f_{i,q}\left(t-R_{m}/c\right)$ and $\psi_{1}=2\pi f_{1}\left(t-R_{1}/c\right)$; then the steering vector of the *i*th subarray can be expressed as

(4) $$a_{i}\left(\Delta f_{i},t,R,\theta \right)=\left[1;e^{-j\Delta \psi_{2}};e^{-j\Delta \psi_{3}};\cdots ;e^{-j\Delta \psi_{Q}}\right]$$

Considering the phase difference between subarrays caused by the wave path difference is given by

(5) $$\phi_{i}\left(\theta \right)=\left(i-1\right)2\pi Qd\sin\left(\theta \right)/\lambda_{0}$$

Therefore, the steering vector of the uniform continuous subarray-based FDA can be expressed as

(6) $$b\left(\left\{\Delta f_{i}\right\},t,R,\theta \right)=\left[\alpha_{1}\left(\theta \right)a_{1}\left(\Delta f_{1},t,R,\theta \right);\cdots ;\alpha_{K}\left(\theta \right)a_{K}\left(\Delta f_{K},t,R,\theta \right)\right]$$

where $b\left(\left\{\Delta f_{i}\right\},t,R,\theta \right)\in {\mathbb{C}}^{M\times 1}$ denotes the steering vector of the whole array, and $\alpha_{i}\left(\theta \right)=e^{j\phi_{i}\left(\theta \right)}$ denotes the inter-subarray phase factor.

Assuming the far-field target is $\left(R_{0},\theta_{0}\right)$, the corresponding weight vector can be constructed as

(7) $$w\left(\Delta f_{i},t,R_{0},\theta_{0}\right)=w_{i}\left(\Delta f_{i},t,R_{0},\theta_{0}\right)⊙\beta \left(\theta_{0}\right)$$

where the operator ⊙ denotes the Hadamard product, $w_{i}\left(\Delta f_{i},t,R_{0},\theta_{0}\right)\in {\mathbb{C}}^{M\times 1}$ and $\beta \left(\theta_{0}\right)\in {\mathbb{C}}^{M\times 1}$ denote the intra-subarray and inter-subarray weight vectors, respectively, given by

(8) $$w_{i}\left(\Delta f_{i},t,R_{0},\theta_{0}\right)={\left[w_{1}^{T}\left(\Delta f_{1},t,R_{0},\theta_{0}\right),\cdots ,w_{K}^{T}\left(\Delta f_{K},t,R_{0},\theta_{0}\right)\right]}^{T}$$

(9) $$\beta \left(\theta_{0}\right)={\left[\beta_{1}1_{Q}^{T},\cdots ,\beta_{K}1_{Q}^{T}\right]}^{T}.$$

Here, the ^T^ denotes transpose, $w_{i}\left(\Delta f_{i},t,R_{0},\theta_{0}\right)=a_{i}\left(\Delta f_{i},t,R,\theta \right){|}_{\left(R,\theta \right)=\left(R_{0},\theta_{0}\right)}$ and $\beta_{i}=\alpha_{i}\left(\theta \right){|}_{\theta =\theta_{0}}$ represent the weight vector of the *i*th subarray and the inter-subarray coefficient, respectively; $1_{Q}^{T}$ denotes *Q*-dimensional all-ones vector.

Accordingly, for uniform continuous subarray-based FDA, the beampattern function with the mainlobe steered toward the target $\left(R_{0},\theta_{0}\right)$ can be expressed as

(10) $$\begin{array}{cc} E\left(\Delta f_{i},t,R_{0},\theta_{0}\right) & =w{\left(\Delta f_{i},t,R_{0},\theta_{0}\right)}^{H}b\left(\Delta f_{i},t,R,\theta \right)\\ & =\sum_{i=1}^{N}\beta_{i}^{*}\alpha_{i}\left(\theta \right)w_{i}^{H}a_{i}\left(\Delta f_{i},t,R,\theta \right) \end{array}$$

From Equation (10), the beampattern function is time-dependent. Therefore, the joint transmit–receive signal model can overcome the inherent time-varying property of the FDA [49]. The signal radiated by the *q*th element of the *i*th subarray, reflected by the far-field target $\left(R,\theta \right)$ and received by the *n*th receive element, can be written as

(11) $$s_{n,\left(i,q\right)}\left(t,R,\theta \right)=\exp\left\{j\left[2\pi f_{i,q}\left(t-\frac{R_{i,q}+R_{n}}{c}\right)\right]\right\}$$

Since the receive array is not partitioned into subarrays and the numbers of transmit and receive elements are equal, the distance from the *n*th receive element to the far-field target is $R_{n}=R-\left(n-1\right)d\sin\left(\theta \right),n=1,\cdots ,N$. Hence, the output of the *n*th receive channel is the sum of all transmit signals.

(12) $$g_{n}\left(t,R,\theta \right)=\sum_{i=1}^{N}\sum_{q=1}^{Q}s_{n,\left(i,q\right)}\left(t,R,\theta \right)$$

**Figure 2:**
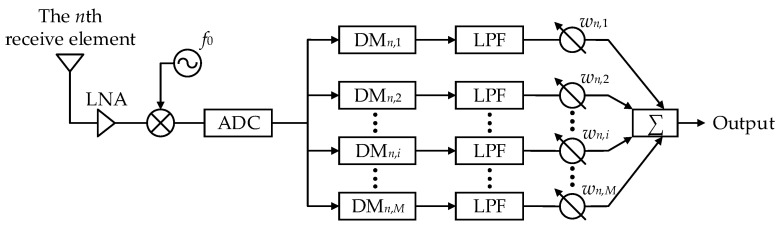
Receiving processing chain of the *n*th receive element.

From Equation (12), the time-varying terms persist, and signal separation has not yet been completed. Figure 2 presents the block diagram of the signal-processing chain for the *n*th receive element, based on the signal model that is derived and analyzed.

As depicted in Figure 2, the signal received by the *n*th receive element is first amplified by a low-noise amplifier (LNA) and then mixed by a mixer driven by a local frequency $f_{0}$. The mixed signal is subsequently sampled by an ADC. Next, a set of digital mixers (DMs) with bandwidth $\Delta f_{i,q}$ is applied to compensate for the time-varying terms introduced by the FO. The signal then passes through a low-pass filter (LPF) and undergoes complex weighting, so that the output of the receive processing chain can be written as

(13) $${\bar{g}}_{n}\left(R,\theta \right)=\sum_{n=1}^{N}\omega_{n,h}{\hat{g}}_{n,h}\left(R,\theta \right)$$

where h=1,2,⋯,M denotes the signal-processing path, $\omega_{n,h}$ denotes the weighting coefficient, and ${\hat{g}}_{n,h}\left(R,\theta \right)=h_{n,h}\left(t\right)*g_{n,h}\left(t,R,\theta \right)$ denotes the signal after LPF.

Consequently, the transmit–receive array factor of the uniform continuous subarray-based FDA can be further expressed as

(14) $$AF_{T-R}\left(R,\theta \right)=\sum_{n=1}^{N}\xi_{n}{\bar{g}}_{n}\left(R,\theta \right)=\sum_{n=1}^{N}\sum_{h=1}^{M}\mu_{n,h}{\hat{g}}_{n,h}\left(R,\theta \right)$$

where $\mu_{n,h}=\xi_{n}\omega_{n,h}$, with $\xi_{n}$ denoting the weight of the *n*th receive element. This transmit–receive signal model will be employed in Section 3 for the beampattern optimization of the subarray-based FDA.

### 2.2. Transmit–Receive Signal Model of Nonuniform and Discontinuous Subarray-Based FDA

**Figure 3:**
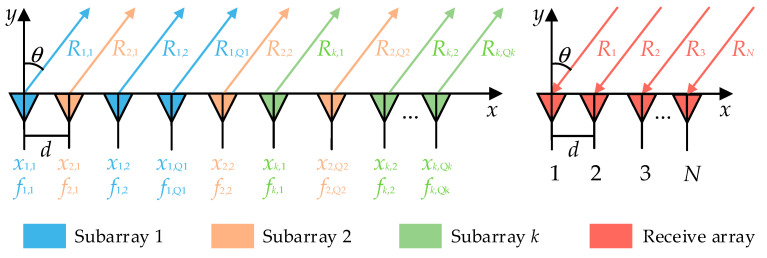
Configuration of the nonuniform and discontinuous subarray-based FDA.

As shown in Figure 3, a collocated transmit-receive linear-FDA consisting of *M* transmit elements and *N* receive elements with element spacing of d is considered. The transmit array is partitioned into K nonuniform and discontinuous subarrays $\left(K<M\right)$, and the number of elements in each subarray as well as their positions may differ.

Since the subarrays are arranged nonuniformly and discontinuously, define the index of the *q*th element belonging to subarray *k* in the array as $x_{k,q}$; set $Q_{k}$ to denote the number of elements in subarray $\sum_{k=1}^{K}Q_{k}=M$. The index set of elements contained in the *k*th subarray is then $\Omega_{k}=\left[x_{k,1},x_{k,2},\cdots ,x_{k,Q_{k}}\right]$. The frequency of the *q*th element in the *k*th subarray can be expressed as

(15) $$f_{k,q}=f_{0}+\left(q-1\right)\Delta f_{k}\;\;\;\;\;q=1,2,\ldots ,Q_{k}\;\;\;\;\;k=1,2,\ldots ,K$$

The overall geometry remains a uniform linear array. The wave path from this element to the far-field target $\left(R,\theta \right)$ is given by

(16) $$R_{k,q}=R-\left(x_{k,q}-1\right)d\sin\left(\theta \right)$$

Accordingly, Equation (3) is rewritten as

(17) $$\Delta \psi_{x_{k,q}}=\psi_{x_{k,q}}-\psi_{1}=2\pi \left[\left(q-1\right)\Delta f_{k}\left(t-\frac{R}{c}\right)-\frac{f_{0}\left(x_{k,q}-1\right)d\sin\left(\theta \right)}{c}\right]$$

The steering vector of the kth subarray can thus be expressed as

(18) $$a_{k}\left(\Delta f_{k},t,R,\theta \right)=\left[1;e^{-j\Delta \psi_{k,2}};\cdots ;e^{-j\Delta \psi_{k,q}}\right]$$

It is worth noting that $a_{k}$ of each subarray already embodies the intra-subarray phase relations induced by the spatial distribution of its elements, and the subarray geometry is irregular. Hence, a fixed inter-subarray phase term need not be included when forming the overall vector. Accordingly, the steering vector of the nonuniform and discontinuous subarray-based FDA can be expressed as

(19) $$b\left(\Delta f_{k},t,R,\theta \right)=\left[a_{1}\left(\Delta f_{1},t,R,\theta \right);\cdots ;a_{K}\left(\Delta f_{K},t,R,\theta \right)\right]$$

Thus, the beampattern function with the mainlobe steered toward the target $\left(R_{0},\theta_{0}\right)$ can be expressed as

(20) $$\begin{array}{cc} E\left(\Delta f_{k},t,R_{0},\theta_{0}\right) & =w{\left(\Delta f_{k},t,R_{0},\theta_{0}\right)}^{H}b\left(\Delta f_{k},t,R,\theta \right)\\ & =\sum_{k=1}^{K}w_{k}^{H}a_{k}\left(\Delta f_{k},t,R,\theta \right) \end{array}$$

Based on the transmit–receive signal model presented in the previous section, the array factor of the nonuniform and discontinuous subarray-based FDA can be expressed as

(21) $$AF_{T-R}\left(\Delta f_{k},R,\theta \right)=\sum_{n=1}^{N}\xi_{n}{\bar{g}}_{n}\left(\Delta f_{k},R,\theta \right)=\sum_{n=1}^{N}\sum_{h=1}^{M}\mu_{n,h}{\hat{g}}_{n,h}\left(\Delta f_{k},R,\theta \right)$$

### 2.3. Theoretical Analysis of Mainlobe Width of Frequency Diversity Array

For the linear-FDA, the fundamental array factor can be expressed as follows:

(22) $$\begin{array}{cc} AF\left(R,\theta ,t\right) & =\sum_{m=1}^{M}e^{j2\pi \left(m-1\right)\left[\Delta f\left(t-\frac{R}{c}\right)+\frac{f_{0}d\sin\left(\theta \right)}{c}\right]}\\ & =e^{j\pi \left(M-1\right)\left[\Delta f\left(t-\frac{R}{c}\right)+\frac{f_{0}d\sin\left(\theta \right)}{c}\right]}\frac{\sin\left[\pi M\left(\Delta f\left(t-\frac{R}{c}\right)+\frac{f_{0}d\sin\left(\theta \right)}{c}\right)\right]}{\sin\left[\pi \left(\Delta f\left(t-\frac{R}{c}\right)+\frac{f_{0}d\sin\left(\theta \right)}{c}\right)\right]} \end{array}$$

Based on the above expression, the null-to-null bandwidth formulas in the range and angle dimensions can be derived as follows:

(23) $$BW_{R}=\frac{2c}{M\Delta f}$$

(24) $$BW_{\theta }=\arcsin\left(\frac{2c}{Mf_{0}d}\right)$$

It can be seen from Equations (23) and (24) that the mainlobe width in range dimension is determined by the *M* and Δ*f*, while the mainlobe width in angle dimension depends on *M*, *f*_0_, and *d*. In the proposed subarray partitioning method, Δ*f* is jointly determined by the element position and the subarray to which the element belongs. This indicates that the subarray partitioning structure dictates the distribution of FO. Therefore, by optimizing the subarray partitioning structure, the distribution can be adjusted, thereby reducing the mainlobe width in range dimension and improving range resolution. It is worth noting that since the mainlobe width in angle dimension is only related to *M*, *f*_0_, and *d*, and these parameters remain unchanged in the proposed subarray partitioning scheme, the mainlobe width in angle dimension does not change. This means that the proposed method can achieve improved range resolution while maintaining the same angle resolution.

## 3. Nonuniform and Discontinuous Subarray Partitioning Method Based on Nested Optimization Algorithm

In this section, the nonuniform and discontinuous subarray partitioning method of linear-FDA is investigated on the basis of the signal model established in Section 2. Specifically, Section 3.1 introduces a scheme based on the K-means++ clustering algorithm, where clustering of element amplitudes yields nonuniform and noncontiguous subarray structure. Building on this foundation, Section 3.2 develops a nested optimization algorithm that integrates the BA with the K-means++ clustering algorithm to jointly optimize subarray structure and element amplitudes, with the goal of enhancing beampattern performance in range and angle dimension.

### 3.1. Nonuniform and Discontinuous Subarray Partitioning Method Based on K-Means++ Clustering Algorithm

In this subsection, the subarray partitioning problem is reformulated as an unsupervised clustering optimization based on element characteristics. Specifically, array elements are grouped according to the similarity of their element amplitudes, enabling adaptive design of the subarray structure. The K-means clustering algorithm [50] is widely used for its simplicity and efficiency. However, its results depend heavily on the random selection of initial cluster centers, and it is prone to converge to a local optimal solution. To address this problem, the K-means++ clustering algorithm is adopted for subarray partitioning, which improves the initialization strategy so that the centers are more widely dispersed in the feature space, thereby enhancing optimization quality and stability.

Assume the linear-FDA with M transmit elements is partitioned into K subarrays. Set $a=\left[a_{1},a_{2},\cdots ,a_{M}\right]$ to denote the element amplitudes and take it as the feature vector. The goal of K-means++ clustering algorithm is to cluster the M transmit elements into the specified K clusters based on inter-element similarity, with each element belonging only to the cluster whose centroid is nearest.

Specifically, one value is randomly selected from the element amplitude a as the initial cluster center $c_{k}$. For each element amplitude $a_{i}$, the squared distance to the nearest already-selected cluster center is computed as $D{\left(a_{i}\right)}^{2}$.

(25) $$D{\left(a_{i}\right)}^{2}=\min{\left\|a_{i}-c_{k}\right\|}^{2}$$

where each element amplitude is chosen as the next cluster center with a probability of $P\left(a_{i}\right)$, and thus the next cluster center is selected according to the roulette wheel selection method. $P\left(a_{i}\right)$ can be expressed as

(26) $$P\left(a_{i}\right)=\frac{D{\left(a_{m}\right)}^{2}}{\sum_{j=1}^{M}D{\left(a_{j}\right)}^{2}}$$

This probability distribution makes it more likely that the next center lies in a region far from the existing centers, thereby spreading the initial centers more broadly in the feature space. The procedure continues until K initial centers $C=\left[c_{1},c_{2},\cdots ,c_{k}\right]$ are obtained. For each element amplitude $a_{m}$, compute the Euclidean distance to all centers and assign it to the cluster with the minimum distance. The *k*th cluster, i.e., subarray $\Omega_{k}$, is composed of the elements that satisfy the following condition.

(27) $$\Omega_{k}=\left\{a_{m}:\left|a_{m}-c_{k}\right|\leq \left|a_{m}-c_{j}\right|,\forall j\neq k\right\}$$

The above steps are based on element amplitude similarity, partitioning the entire array into K subarrays that are nonuniform and discontinuous. The center of each cluster is then updated as the mean of the element amplitudes currently assigned to that cluster.

(28) $$c_{k}=\frac{1}{\left|\Omega_{k}\right|}\sum_{a_{m}\in \Omega_{k}}a_{m}$$

Iterating in this manner, the final output is the optimized subarray partition $X=\left\{\Omega_{1},\Omega_{2},\cdots ,\Omega_{k}\right\}$.

For the problem of nonuniform and discontinuous subarray-based FDA addressed in this section, the goal is to find the optimal subarray configuration X such that the generated beampattern is, in a certain sense, the best approximation to the desired beampattern. The fitness function is defined as the square of a weighted error between the current and desired beampatterns with respect to mainlobe width and PSLL in range dimension.

(29) $$F\left(X\right)=\eta_{1}{\left(PSLL_{r}-PSLL_{ref}\right)}^{2}+\eta_{2}{\left(HPBW_{r}-HPBW_{ref}\right)}^{2}$$

where $\eta_{1}$ and $\eta_{2}$ are weighting coefficients; *PSLL_r_* and *HPBW_r_* denote the simulated PSLL and half-power beam width (HPBW) in range dimension, respectively. $PSLL_{ref}$ and $HPBW_{ref}$ denote the desired PSLL and HPBW in range dimension.

Therefore, the optimization process can be described as

(30) $$\begin{array}{cc} \min & F\left(X\right) \end{array}$$

(31) $$\begin{array}{cc} s.t & \cup_{k=1}^{K}\Omega_{k} \end{array}=\left\{1,2,\cdots ,M\right\}$$

(32) $$\begin{array}{cc} \Omega_{i}\cap \Omega_{j}=\emptyset & \forall i\neq j \end{array}$$

Under the imposed constraints, the subarray partitioning structure is iteratively updated via the K-means++ clustering algorithm to minimize the fitness function, which can enable FDA to generate the narrowest HPBW and the lowest PSLL in range dimension. The detailed algorithmic procedure is as follows (Algorithm 1).
**Algorithm 1.** Optimization procedure for subarray partitioning based on K-means++ clustering algorithm$\mathrm{Input}:\;M,\;K,\;f_{0},\;\;\Delta f_{k},\;a,\;P$$\mathrm{Output}:\;X=\left\{\Omega_{1},\;\Omega_{2},\;\cdots ,\;\Omega_{k}\right\}$$,\;F\left(X\right)$Step 1. Initialize the iteration counter p=1$.\;\mathrm{Randomly}\;\mathrm{select}\;\mathrm{the}\;\mathrm{first}\;\mathrm{cluster}\;\mathrm{center}\;c_{1}$ from the element amplitude set.$\mathrm{Step}\;2.\;\mathrm{For}\;\mathrm{each}\;\mathrm{unselected}\;\mathrm{element}\;\mathrm{amplitude}\;a_{m}$$,\;\mathrm{compute}\;\mathrm{the}\;\mathrm{squared}\;\mathrm{distance}\;\mathrm{to}\;\mathrm{the}\;\mathrm{nearest}\;\mathrm{selected}\;\mathrm{center},\;\mathrm{and}\;\mathrm{select}\;\mathrm{the}\;\mathrm{next}\;\mathrm{center}\;c_{k}$ according to (26). Repeat until K$\;\mathrm{initial}\;\mathrm{centers}\;\left\{c_{1},\;c_{2},\;\cdots ,c_{K}\right\}$ are obtained.$\mathrm{Step}\;3.\;\mathrm{According}\;\mathrm{to}\;(27),\;\mathrm{assign}\;\mathrm{the}\;\mathrm{elements}\;\mathrm{to}\;\mathrm{the}\;\mathrm{nearest}\;\mathrm{cluster},\;\mathrm{forming}\;\mathrm{the}\;\mathrm{subarray}\;\mathrm{partition}\;\Omega_{k}$.Step 4. Recompute each cluster center using (28).$\mathrm{Step}\;5.\;\mathrm{According}\;\mathrm{to}\;(29),\;\mathrm{calculate}\;F\left(X\right)$ of the current subarray partition.Step 6. Update Ito p=p+1$,\;\mathrm{repeat}\;\mathrm{steps}\;3\;\mathrm{to}\;5\;\mathrm{until}\;\mathrm{the}\;\mathrm{maximum}\;\mathrm{iteration}\;\mathrm{count}\;\mathrm{is}\;\mathrm{reached}\;\mathrm{or}\;\mathrm{the}\;F\left(X\right)$ converges.$\mathrm{Step}\;7.\;\mathrm{Determine}\;\mathrm{the}\;\mathrm{optimal}\;\mathrm{subarray}\;\mathrm{configuration}\;X=\left\{\Omega_{1},\;\Omega_{2},\;\cdots ,\Omega_{k}\right\}$$\;\mathrm{and}\;\mathrm{its}\;\mathrm{corresponding}\;\mathrm{optimal}\;F\left(X\right)$.

### 3.2. Nonuniform and Discontinuous Subarray Partitioning Method Based on BA and K-Means++ Clustering Algorithm

The beampattern of nonuniform and noncontiguous subarray-FDA with the above method depends on the initial element amplitudes; however, these element amplitudes are random. To further improve beamforming performance, this subsection proposes a nested optimization algorithm that integrates BA and the K-means++ clustering algorithm. This algorithm jointly optimizes the subarray structure and the element amplitudes, combining the global search capability of the BA with the efficient clustering property of the K-means++ clustering algorithm, so as to realize global optimization of beampattern performance in the range–angle dimension.

BA is a swarm-intelligence optimization algorithm inspired by bat echolocation behavior [51]. In this algorithm, each bat represents a candidate solution in the search space, and the optimal solution is sought by simulating bat flight, echolocation, and prey-capturing behaviors. BA offers strong global search capability and fast convergence, making it suitable for joint optimization of subarray configuration and element amplitudes in combination with the K-means++ clustering algorithm. Specifically, first initialize *I* bats within the feasible domain, and define the position of each bat as $a=\left[a_{1},a_{2},\cdots ,a_{M}\right]$, where $a_{m}$ denotes the element amplitude of the *m*th array element over the global domain. During the *t*th iteration, the frequency $Fr_{i}$, velocity $v_{i}$, and position $a_{i}$ of the *i*th bat are updated according to the following equations.

(33) $$Fr_{i}=Fr_{\min}+\left(Fr_{\max}-Fr_{\min}\right)\delta$$

(34) $$v_{i}^{t+1}=v_{i}^{t}+Fr_{i}\left(a_{i}^{t}-a_{g}^{t}\right)$$

(35) $$a_{i}^{t+1}=a_{i}^{t}+v_{i}^{t+1}$$

where $Fr_{\max}$ and $Fr_{\min}$ denote the predefined upper and lower bounds of the frequency, respectively. $\delta \in \left[0,1\right]$ is a uniformly distributed random variable; $a_{g}^{t}$ denotes the global best position of all bats at iteration *t*. As the iterations proceed, the loudness $A_{i}$ and the rate $r_{i}$ are updated as follows

(36) $$A_{i}^{t+1}=\varsigma A_{i}^{t}$$

(37) $$r_{i}^{t+1}=r_{i}^{1}\left[1-\exp\left(-\gamma t\right)\right]$$

where $\varsigma \in \left(0,1\right)$ and γ>0 are predefined constants to guarantee $\underset{t\to \infty }{\lim}A_{i}=0$ and $\underset{t\to \infty }{\lim}r_{i}=r_{i}^{1}$ which indicates that an optimal solution is found and the iteration can be stopped.

It is worth noting that $A_{i}$ and $r_{i}$ are updated only after the position of a bat has been improved. For each $a_{m}$ optimized by BA, the K-means++ clustering algorithm is used to perform subarray partitioning: exploiting the similarity of element amplitudes, the array is dynamically divided into K nonuniform and noncontiguous subarrays, with the partitioning procedure given by Equations (25)–(28). Accordingly, the fitness function can be rewritten as

(38) $$F\left(a,X\right)=\eta_{1}{\left(PSLL_{r}-PSLL_{ref}\right)}^{2}+\eta_{2}{\left(HPBW_{r}-HPBW_{ref}\right)}^{2}$$

The optimization problem is formulated as

(39) $$\begin{array}{cc} \min & F\left(a,X\right) \end{array}$$

(40) $$\begin{array}{cc} s.t & \cup_{k=1}^{K}\Omega_{k} \end{array}=\left\{1,2,\cdots ,M\right\}$$

(41) $$\begin{array}{cc} \Omega_{i}\cap \Omega_{j}=\emptyset & \forall i\neq j \end{array}$$

(42) $$a\in \left(0,1\right]$$

**Figure 4:**
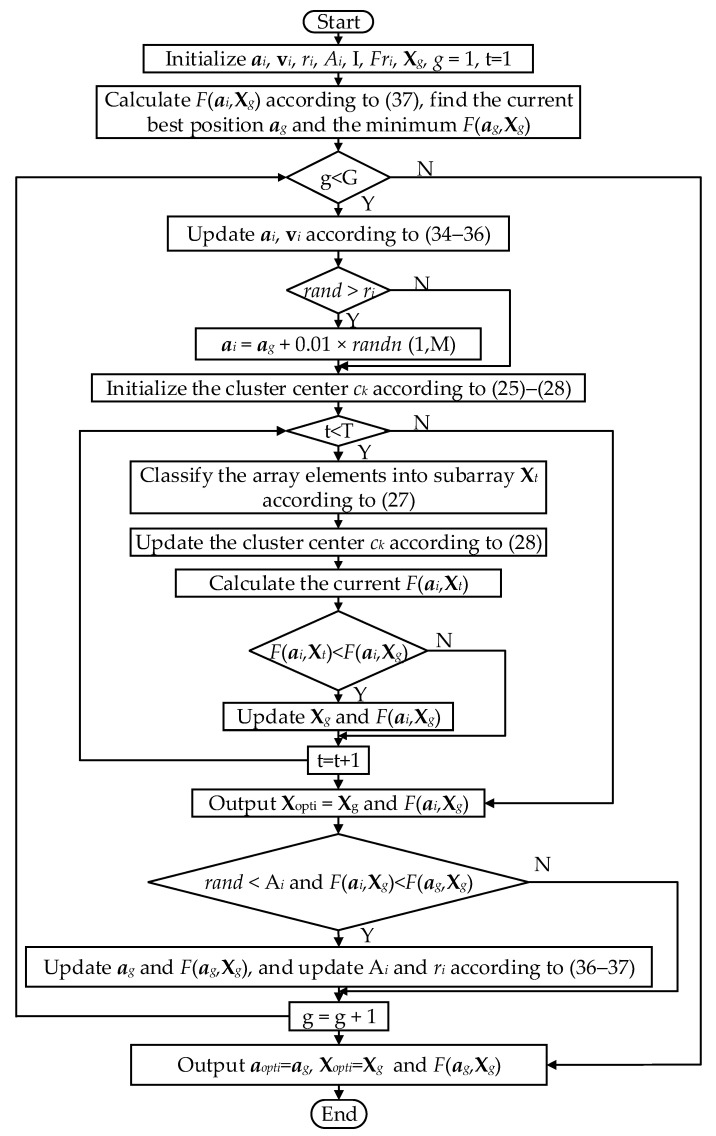
Detailed flowchart of Algorithm 2.

The joint optimization algorithm adopts a nested-loop architecture. The outer loop employs BA to optimize the element amplitudes, and the inner loop applies the K-means++ clustering algorithm to repartition the subarray configuration according to the current element amplitudes. The two procedures operate in a nested, coordinated manner to minimize $F\left(a,X\right)$, thereby yielding a beampattern with the narrowest HPBW and the lowest PSLL in range dimension. The detailed algorithmic flowchart is as follows (Figure 4).

## 4. Numerical Results and Discussion

In this section, the effectiveness of the proposed subarray partitioning methods is analyzed in detail. Firstly, under the same subarray configuration, linear FOs and nonlinear FOs are applied separately to systematically assess the impact of FO on beampattern performance of the uniform continuous subarray-based FDA. Then, a nonuniform discontinuous subarray partitioning method based on the K-means++ clustering algorithm is investigated; by adaptively clustering according to the similarity of element amplitudes, the spatial constraints of traditional contiguous partitioning are lifted. Finally, a joint optimization algorithm is employed to simultaneously optimize the subarray structure and the element amplitudes; thus, the performance of the subarray-based FDA is further enhanced. The target is located at $\left(R_{0},\theta_{0}\right)=\left(50\;\mathrm{km},0{}^{\circ}\right)$, and the total observation region is defined as $\varpi =\left\{\left(R,\theta \right)|20\;\mathrm{km}\leq R\leq 80\;\mathrm{km},\;-90{}^{\circ}\leq \theta \leq 90{}^{\circ}\right\}$.

### 4.1. Uniform Continuous Subarray-Based FDAs

To systematically investigate the beamforming performance of uniform continuous subarray structures, this subsection divides the FDA with M=N=60 elements into three subarrays and compares the beampatterns obtained with linear FOs and nonlinear FOs. The specific simulation parameters are listed in Table 1.

**Figure 5:**
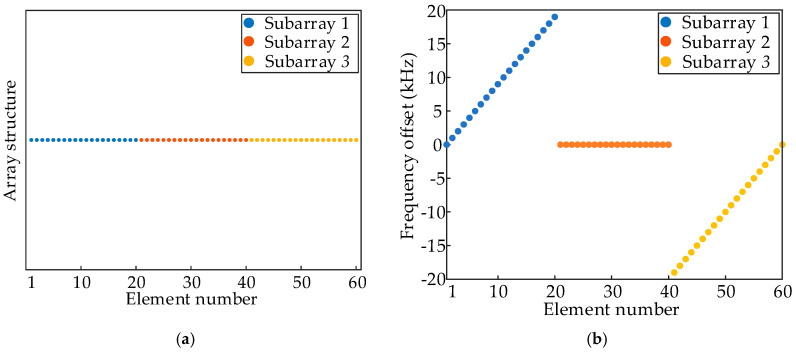
The (**a**) array structure and (**b**) FO distributions of uniform continuous subarray-based FDA.

Under the uniform continuous subarray structure, the M elements are divided into K contiguous subarrays in a spatial order, each containing Q consecutively placed elements, i.e., subarray 1 includes $\left\{1,2,\cdots 20\right\}$, subarray 2 includes $\left\{21,22,\cdots 40\right\}$, and subarray 3 includes $\left\{41,42,\cdots 60\right\}$. The structure of subarray-based FDA is shown in Figure 5a. Different linear FOs are assigned to the subarrays as follows:

(43) $$f_{1,q}=f_{0}+\left(q-1\right)\cdot \Delta f$$

(44) $$f_{2,q}=f_{0}+\left(q-1\right)\cdot 0$$

(45) $$f_{3,q}=f_{0}+\left(q-Q\right)\cdot \Delta f$$

where Δf=1 kHz. The FO distribution is shown in Figure 5b, and the overall FDA exhibits central symmetry in frequency.

**Figure 6:**
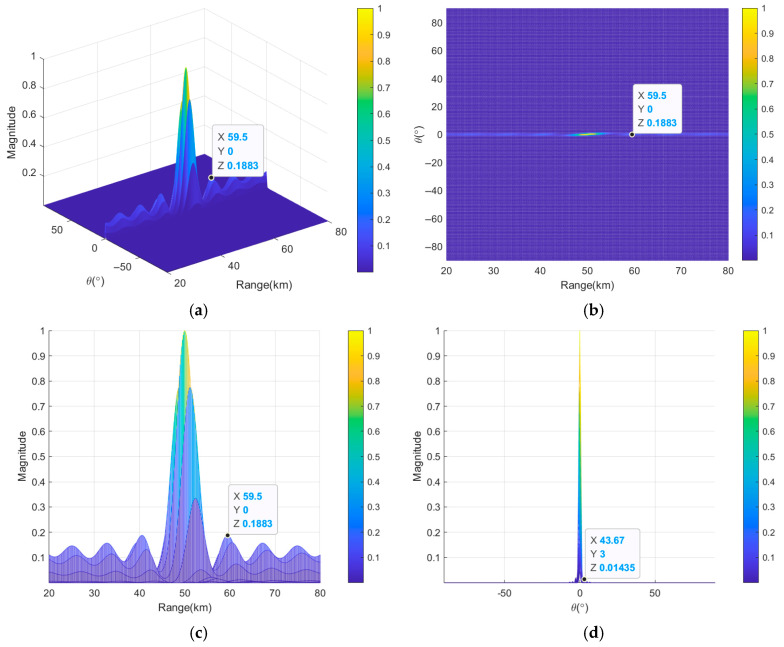
Transmit–receive beampatterns of uniform continuous subarray-based FDA with linear FOs, where (**a**) is in 3D and (**b**–**d**) are the projections in range and angle dimensions, in range dimension and in angle dimension, respectively.

Figure 6 shows the transmit–receive beampatterns of uniform continuous subarray-based FDA with linear FOs. From Figure 6a,b, an energy focus is formed at the target location, but multiple high-energy sidelobe peaks exist in other regions of range–angle dimension. Figure 6c gives the corresponding beampattern projection in range dimension. It can be seen that a relatively sharp mainlobe is formed at the target location, and the *HPBW_r_* is 4.35 km, indicating good beam-focusing capability. However, the *PSLL_r_* is 10log_10_(0.1883) = −7.25 dB, and the sidelobe-suppression capability is insufficient. In practical applications, higher sidelobes lead to the generation of clutter, thereby reducing the accuracy of target detection. Figure 6d provides the beampattern projection in angle dimension. It can be seen that the HPBW is 1.15° and the PSLL is 10log_10_(0.01435) = −18.43 dB. Although a uniform continuous subarray-based FDA with linear FOs can realize beam-focusing performance, the high PSLL problem significantly limits its value in practical engineering applications.

**Figure 7:**
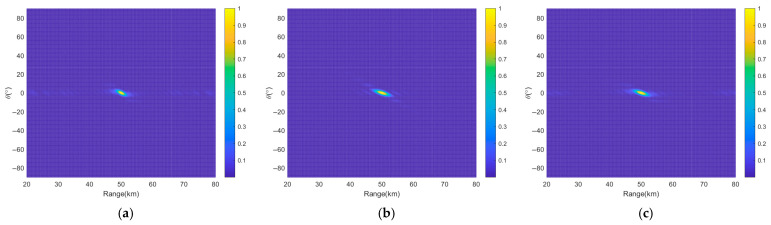
The range–angle projection beampatterns of three nonlinear frequency offsets, where the FO is based on the (**a**) arsinh-function, (**b**) sin-function, (**c**) ln-function, respectively.

When partitioning the array into subarrays, only when all subarrays form mainlobes in the same region and the mainlobes can be superimposed in phase does this region form the mainlobe of the entire array. However, the beampattern of the subarray employing linear FOs inherently suffers from a broad mainlobe and high sidelobe levels. As a result, the synthesized overall beampattern still struggles to meet the requirements in terms of mainlobe width and sidelobe suppression. To address this issue, this paper, based on the paper of nonlinear FOs in reference [52], selects three nonlinear FO schemes with superior performance. These schemes are applied to three subarrays, enabling each subarray to independently form a focused beampattern, as shown in Figure 7. After the superposition of the three focused beampatterns, the overall beampattern exhibits a narrower mainlobe width and lower sidelobe levels. It is worth pointing out that the reason linear FO schemes require a central symmetry FO configuration is that the beampatterns of their subarrays lack inherent focusing capability and must rely on the cross-superposition of beampatterns with different deflection directions to achieve overall focusing. In contrast, each beampattern of subarray with nonlinear Fo possesses good focusing characteristics, allowing focusing to be achieved without the need for a central symmetry configuration. Therefore, the absence of central symmetry does not negatively impact the focusing performance of the proposed method. The definitions of the three nonlinear FOs are as follows:

(46) $$f_{1,q}=f_{0}-\Delta f\cdot \left[3.34\cdot \mathrm{arcsinh}(0.34\cdot q)\right]$$

(47) $$f_{2,q}=f_{0}-\Delta f\cdot \left[9\cdot \sin\left(q/38.8\right)\right]$$

(48) $$f_{3,q}=f_{0}-\Delta f\cdot \ln{\left(q\right)}^{1.5}$$

**Figure 8:**
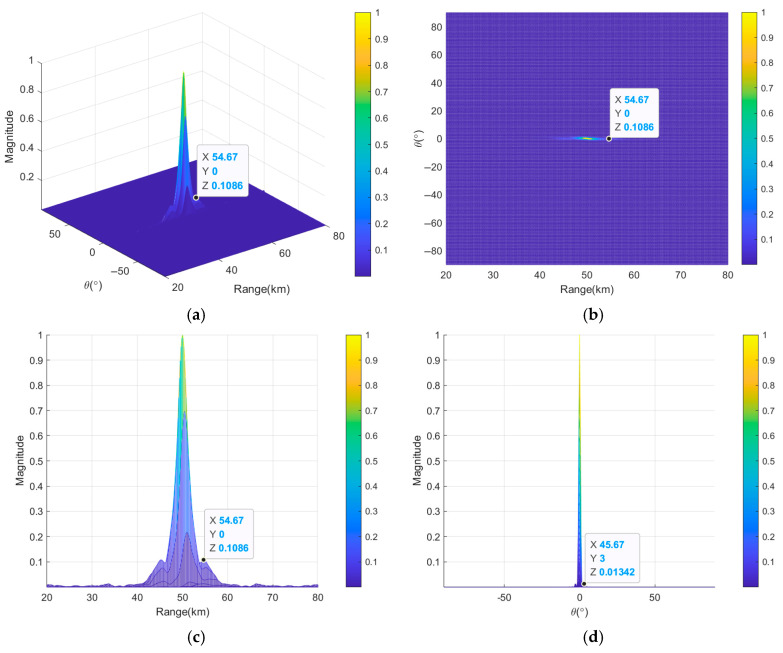
Transmit–receive beampatterns of uniform continuous subarray-based FDAs with nonlinear FOs, where (**a**) is in 3D and (**b**–**d**) are the projections in range and angle dimensions, in range dimension and in angle dimension, respectively.

Figure 8 presents the transmit–receive beampatterns of a uniform continuous subarray-based FDA employing nonlinear FOs. As observed in Figure 8a,b, this subarray-based FDA achieves beam focusing on the target location, with sidelobe suppression in non-target regions. From Figure 8c, it can be clearly observed that the *HPBW_r_* is greatly decreased (from 4.35 km to 2.76 km), and the *PSLL_r_* is reduced by 2.39 dB (from 10log_10_(0.1883) = −7.25 dB to 10log_10_(0.1086) = −9.64 dB). Comparing Figure 8d with Figure 6d, it can be seen that the beampattern projections in angle dimension of the subarray-based FDAs with linear FO and nonlinear FO are identical. In conclusion, compared to the subarray-based FDA with linear FOs, the subarray-based FDA with nonlinear FOs can achieve better performance in terms of range resolution and sidelobe suppression.

**Figure 9:**
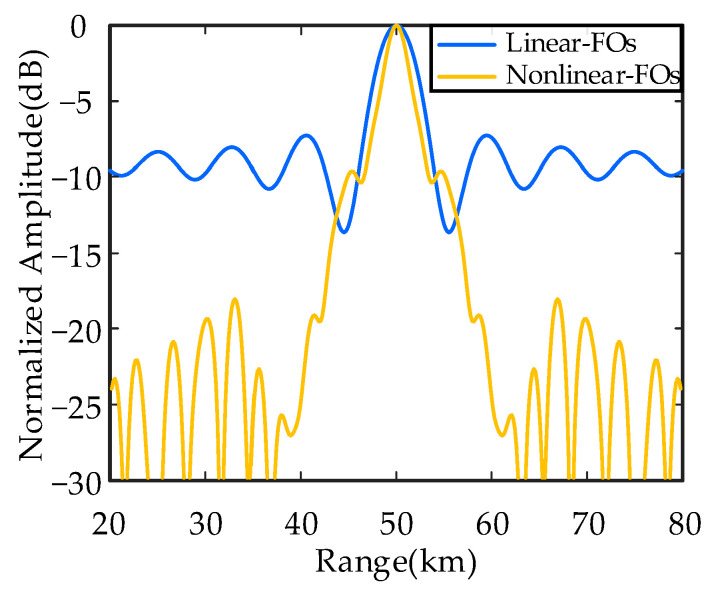
Normalized transmit–receive beampattern profiles of uniform continuous subarray-based FDAs cut at an angle of 0°.

Figure 9 shows the transmit–receive beampattern projection in range dimension of uniform continuous subarray-based FDAs with linear and nonlinear FOs. Table 2 provides detailed comparisons of beampattern performance of the above FDAs. In range dimension, the *HPBW_r_* is 4.35 km of subarray-based FDA with linear FOs, while the FDA with nonlinear FOs narrows it down to 2.76 km, representing a 36.6% reduction. The *PSLL_r_* decreased from 10log_10_(0.1883) = −7.25 dB to 10log_10_(0.1086) = −9.64 dB, achieving a 2.39 dB decrease and approximately 1.7 times greater sidelobe suppression capability. In angle dimension, both subarray-based FDAs demonstrate nearly identical performance. The HPBW in angle dimension (*HPBW_θ_*) remains at 1.15°, while the PSLL in angle dimension (*PSLL_θ_*) are 10log_10_(0.01435) = −18.43 dB and 10log_10_(0.01342) = −18.72 dB, respectively, with a marginal difference of only 0.29 dB. The results indicate that nonlinear FOs not only effectively suppress sidelobes but also enhance range resolution, yielding significantly superior performance compared to linear FOs. This improvement originates from the breaking of the linear dependence between FOs and element spacing by nonlinear FOs, which alleviates range–angle coupling issues and optimizes energy distribution. This advancement lays the groundwork for subsequent optimized designs of nonuniform and discontinuous subarray-based FDAs.

### 4.2. Nonuniform and Discontinuous Subarray-Based FDAs

To further enhance beamforming performance, this subsection investigates two methods to achieve a nonuniform discontinuous subarray-based FDA. Unlike uniform continuous methods where elements are partitioned sequentially according to their spatial positions, the nonuniform discontinuous subarray partitioning method allows elements belonging to the same subarray to be alternately distributed in space. By breaking the constraints of traditional continuous partitioning, this method offers greater design freedom.

The simulation employs the same array configuration as in Section 4.1. Table 3 lists the specific parameters based on the K-means++ clustering algorithm. Among them, *P* is the number of iterations of the algorithm. It should be noted that the initial element amplitudes are random.

**Figure 10:**
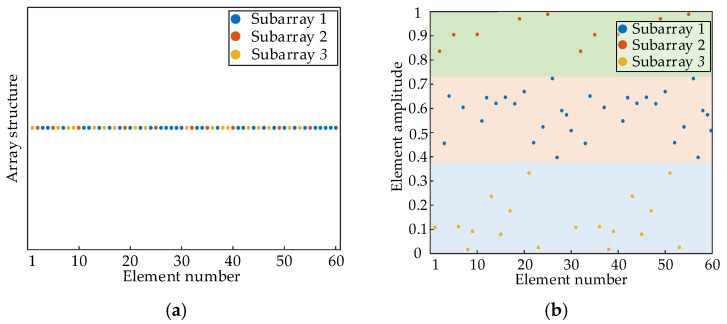
The (**a**) array structure and (**b**) element amplitude distribution of nonuniform and discontinuous subarray-based FDA based on the K-means++ clustering algorithm.

Figure 10a illustrates the optimized subarray partitioning structure based on the K-means++ clustering algorithm, where different colors indicate the affiliation of each subarray. The optimized three subarrays demonstrate significantly discontinuous alternating distribution characteristics, and the number of array elements within each subarray is also different, forming a sharp contrast with the sequential structure of uniform continuous subarray-based FDAs. Figure 10b displays the corresponding element amplitude distribution of array elements, revealing the clustering characteristic of the K-means++ clustering algorithm based on element amplitude similarity. This process automatically groups elements with similar element amplitudes without being constrained by their spatial continuity. The FOs of subarray-based FDAs follow the specifications in Equations (46)–(48).

**Figure 11:**
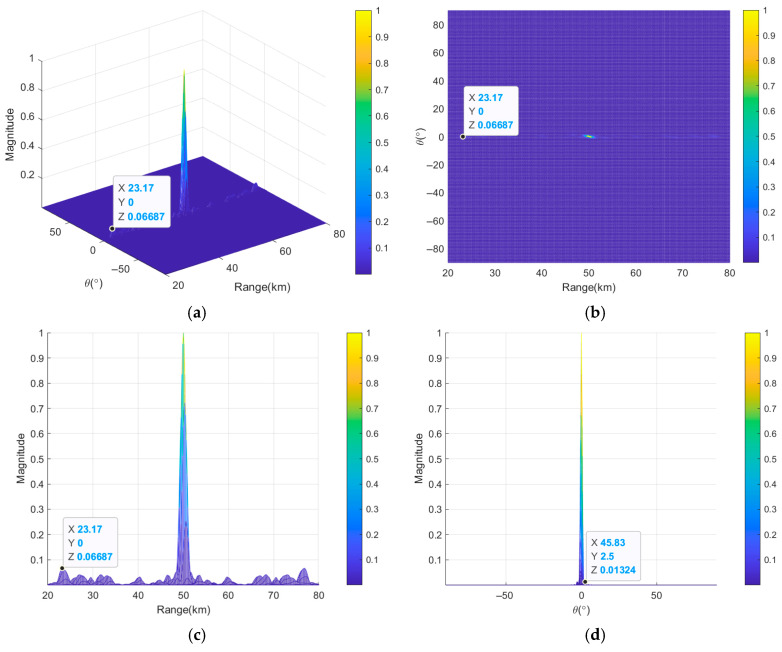
Transmit–receive beampatterns of nonuniform discontinuous subarray-based FDA based on K-means++ clustering algorithm, where (**a**) is in 3D and (**b**–**d**) are the projections in range and angle dimensions, in range dimension and in angle dimension, respectively.

Figure 11 presents the transmit–receive beampatterns of a nonuniform and discontinuous subarray-based FDA employing nonlinear FOs based on the K-means++ clustering algorithm. As observed in Figure 11a,b, this subarray-based FDA results in concentrated beam energy in the target region and sidelobe suppression in non-target regions. Figure 11c reveals that the *HPBW_r_* is narrowed to 1.29 km, and the *PSLL_r_* is reduced to 10log_10_(0.06687) = −11.75 dB, demonstrating superior range resolution and sidelobe suppression compared to the two previously discussed uniform continuous subarray-based FDAs. Compared with Figure 8d, Figure 11d indicates stable performance in angle dimension, with the *HPBW_θ_* remaining at 1.15° and the *PSLL_θ_* at 10log_10_(0.01324) = −18.78 dB, confirming that the nonuniform discontinuous subarray structure does not significantly affect angle beamforming. In summary, these results demonstrate that the combination of the nonuniform discontinuous subarray configuration and nonlinear FOs effectively enhance the beampattern performance in range–angle dimension. This approach not only improves range resolution but also maintains favorable performance in angle dimension, thereby validating the effectiveness of the proposed approach.

In order to obtain the optimal element amplitudes and their corresponding subarray configuration, a nested algorithm to simultaneously optimize both the subarray structure and the element amplitudes are proposed. Specific simulation parameters based on a nested algorithm are listed in Table 4, where *I* represents the population size of the bat algorithm, and *T* and *P* denote the number of iterations of the BA and the K-means++ clustering algorithm, respectively.

**Figure 12:**
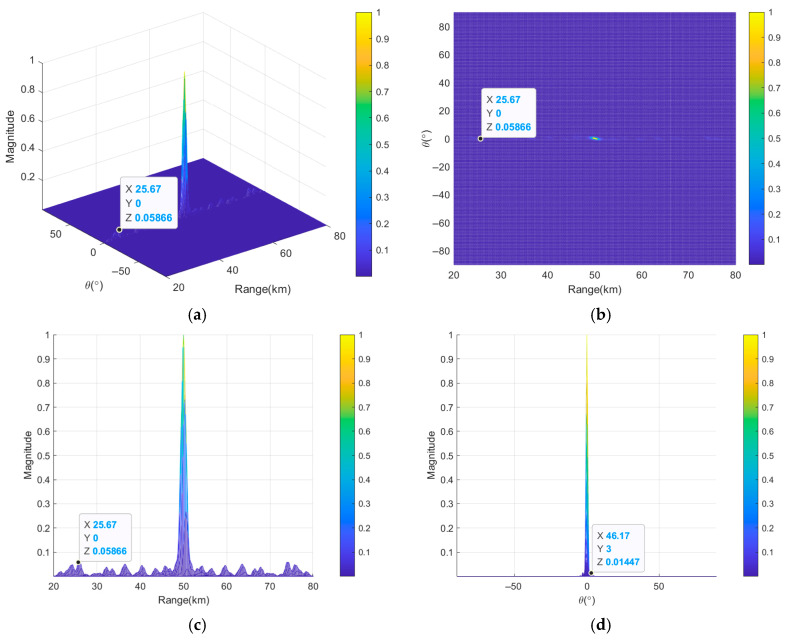
Transmit–receive beampatterns of nonuniform discontinuous subarray-based FDA based on nested algorithm, where (**a**) is in 3D and (**b**–**d**) are the projections in range and angle dimensions, in range dimension and in angle dimension, respectively.

Figure 12 illustrates the transmit–receive beampatterns of nonuniform and discontinuous subarray-based FDA employing nonlinear FOs after joint optimization based on the nested algorithm. It can be clearly seen that the subarray-based FDA after joint optimization can produce a more focused beampattern in Figure 12a,b. Figure 12c shows that the *HPBW_r_* reaches 1.18 km; this means that compared to the 1.29 km achieved without joint optimization, an 8.5% reduction has been achieved. The *PSLL_r_* decreases from 10log_10_(0.06687) = −11.75 dB to 10log_10_(0.05866) = −12.32 dB, indicating enhanced range resolution and sidelobe suppression capability. Figure 12d reveals that the *HPBW_θ_* remains at 1.15° with *PSLL_θ_* of −10log_10_(0.01447) = −18.39 dB, demonstrating that the joint optimization process improves beampattern performance in range dimension without compromising beampattern performance in angle dimension.

**Figure 13:**
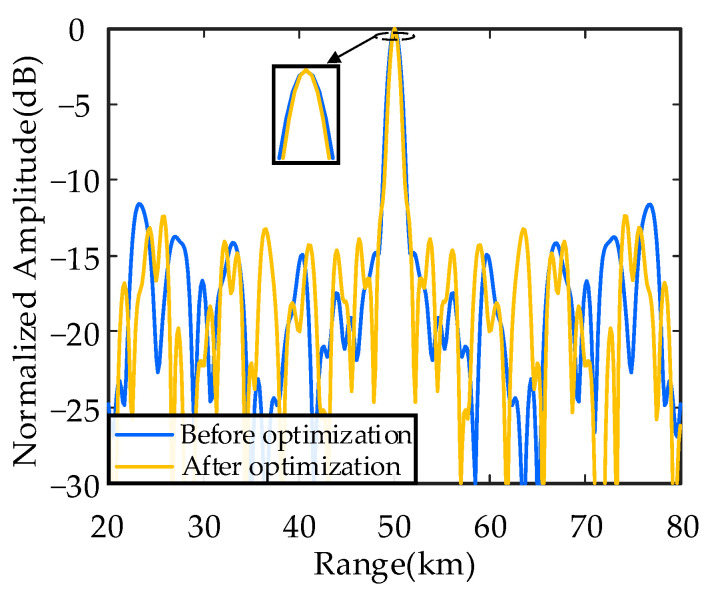
Normalized transmit–receive beampattern profiles of nonuniform discontinuous subarray-based FDAs cut at an angle of 0°.

Figure 13 shows the transmit–receive beampattern projection in range dimension of nonuniform discontinuous subarray-based FDAs under unoptimized and nested-algorithm-optimized conditions. The details are listed in Table 5. The results show that the nested algorithm further improves performance in range dimension. The *HPBW_r_* is reduced from 1.29 km to 1.18 km, while the *PSLL_r_* is decreased from 10log_10_(0.06687) = −11.75 dB to 10log_10_(0.05866) = −12.32 dB. The performance in angle dimension remains generally stable, with the *HPBW_θ_* maintained at 1.15° in both conditions, and *PSLL_θ_* is 10log_10_(0.01324) = −18.78 dB and 10log_10_(0.01447) = −18.39 dB, respectively, differing by only 0.39 dB. These results validate the effectiveness of the nested algorithm, which combines global search in the outer loop and efficient clustering algorithm in the inner loop to achieve co-design of structural parameters and excitation configurations, providing a new approach for enhancement of beampattern performance. Compared with the uniform continuous subarray-based FDA in Section 4.1, the nonuniform discontinuous subarray-based FDAs demonstrate significant performance advantages both before and after optimization.

From a computational complexity perspective, when only the K-means++ clustering algorithm is used for subarray partitioning, its complexity is $O\left(P\cdot M\right)$. In contrast, the nested optimization algorithm has a complexity of $O\left(T\cdot I\cdot P\cdot M\right)$. Thus, while the nested optimization improves range resolution, it comes at the cost of significantly increased computation time. For high-precision design scenarios with adequate computational resources, this additional overhead is acceptable. However, for applications with stringent real-time requirements, the subarray partitioning method based solely on the K-means++ clustering algorithm can be employed to meet the need for timeliness.

**Figure 14:**
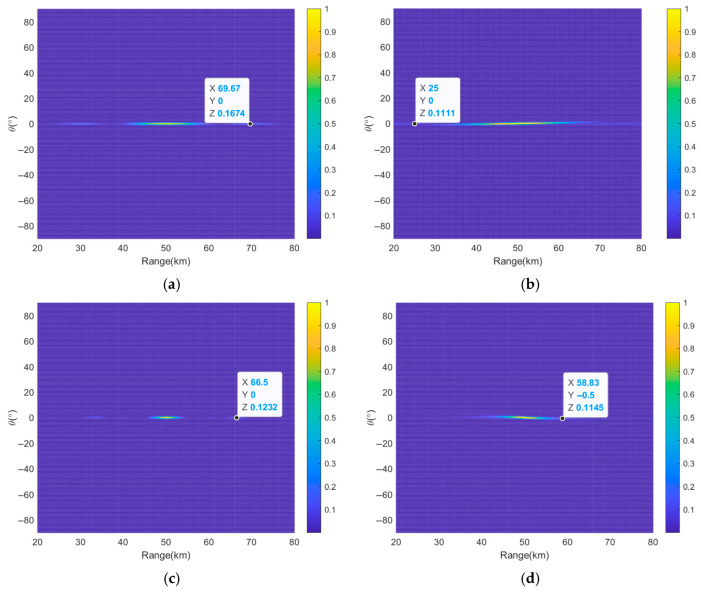
Performance comparison of range–angle projection beampatterns between the proposed method and existing methods, where (**a**) is the centrosymmetric subarray in [33], (**b**) is the uniform continuous subarray in [45], (**c**) is the cross-subarray in [46], and (**d**) is the overlapping subarray in [47], respectively.

To further validate the superiority of the proposed method, Figure 14 and Table 6 provide a performance comparison with several existing FDA subarray-based methods [33,45,46,47] in terms of HPBW and PSLL in both range and angle dimensions. As shown in Figure 14 and Table 6, the proposed method achieves a range HPBW of 1.18 km and a PSLL of −12.32 dB, while also maintaining superior performance in the angle dimension. These results demonstrate that the proposed method outperforms existing methods in terms of range resolution and sidelobe suppression, confirming its effectiveness for high-resolution FDA beamforming.

## 5. Conclusions

FDA boosts the flexibility of beamforming by applying an additional FO across the array aperture; thus, it has great potential applications in target detection. Generally, linear-FDA with linear FO always generates an “S-shaped” transmit beampattern, reducing the accuracy of target detection. This paper focuses on enhancing the range resolution of FDA through subarray-based beamforming method. Compared to the beampatterns of uniform continuous subarray-based FDAs with linear and nonlinear FOs, the superiority of nonlinear FO is verified. Simulation results demonstrate that subarray-based FDA with nonlinear FOs can achieve a lower PSLL, and the *HPBW_r_* is decreased by 1.41 km. For this design approach, the nonuniform discontinuous subarray-based FDA through the K-means++ clustering algorithm is further studied. By breaking traditional continuous partitioning constraints and implementing different types of nonlinear FOs across different subarrays, significant enhancement in range resolution can be achieved. Furthermore, a joint optimization algorithm integrating the BA and K-means++ clustering algorithm is designed to achieve co-design of subarray structure and element amplitudes. Both PSLL and HPBW in range dimension are incorporated into the optimization problem. Simulation results show that after optimization, the *HPBW_r_* is further reduced to 1.18 km and the *PSLL_r_* is decreased to −12.32 dB. Note that the nonuniform discontinuous subarray-based FDAs with nonlinear FOs did not widen the mainlobe width in angle dimension. It can be concluded that the proposed methods can effectively resolve the range–angle coupling issue and provide superior performance in range resolution, providing a feasible approach for enhancing practical performance in target detection and anti-jamming scenarios.

## Figures and Tables

**Table 1:** Simulation parameters of uniform continuous subarray-based FDA.

Parameters	Values	Parameters	Values	Parameters	Values
$f_{0}$	10 GHz	M	60	N	60
d	$\lambda_{0}/2$	K	3	Q	20

**Table 2:** Performance comparisons between linear FOs and nonlinear FOs of uniform continuous subarray-based FDAs.

Type-FO	*HPBW_r_* (km)	*PSLL_r_* (dB)	*HPBW_θ_* (°)	*PSLL_θ_* (dB)
Linear-FOs	4.35	−7.25	1.15	−18.43
Nonlinear-FOs	2.76	−9.64	1.15	−18.72
Relative Variation	−1.59	−2.39	0	−0.35

**Table 3:** Simulation parameters of nonuniform discontinuous subarray-based FDA based on K-means++ clustering algorithm.

Parameters	Values	Parameters	Values	Parameters	Values
M	60	N	60	$f_{0}$	10 GHz
K	3	$HPBW_{ref}$	2	$PSLL_{ref}$	0.07
$\eta_{1}$	1	$\eta_{2}$	1	P	100

**Table 4:** Simulation parameters of nonuniform discontinuous subarray-based FDA based on nested algorithm.

Parameters	Values	Parameters	Values	Parameters	Values
M	60	N	60	K	3
$f_{0}$	10 GHz	$PSLL_{ref}$	0.07	$HPBW_{ref}$	2
I	50	T	100	P	100
$\eta_{1}$	1	$\eta_{2}$	1	$Fr_{min}$	0
$Fr_{max}$	2	$r^{1}$	0.9	$A^{1}$	0.9
γ	0.8	ς	0.8		

**Table 5:** Performance comparison between before and after optimization.

	*HPBW_r_* (km)	*PSLL_r_* (dB)	*HPBW_θ_* (°)	*PSLL_θ_* (dB)
Before optimization	1.29	−11.75	1.15	−18.78
After optimization	1.18	−12.32	1.15	−18.39
Relative Variation	−0.11	−0.57	0	+0.39

**Table 6:** Performance comparison of the proposed method with existing methods.

Reference	*HPBW_r_* (km)	*PSLL_r_* (dB)	*HPBW_θ_* (°)	*PSLL_θ_* (dB)
[33]	11.58	−7.76	1.15	−18.49
[45]	15.5	−9.54	1.15	−16.85
[46]	4.51	−9.09	1.15	−22.07
[47]	6.32	−9.41	1.22	−15.58
Pro.	1.18	−12.32	1.15	−18.39

## Data Availability

The original contributions presented in this study are included in the article. Further inquiries can be directed to the corresponding authors.
